# Biochemistry of Aminoacyl tRNA Synthetase and tRNAs and Their Engineering for Cell-Free and Synthetic Cell Applications

**DOI:** 10.3389/fbioe.2022.918659

**Published:** 2022-07-01

**Authors:** Ragunathan Bava Ganesh, Sebastian J. Maerkl

**Affiliations:** School of Engineering, Institute of Bioengineering, École Polytechnique Fédérale de Lausanne, Lausanne, Switzerland

**Keywords:** AARS, tRNA, genetic code, synthetic biology, cell-free systems, molecular engineering

## Abstract

Cell-free biology is increasingly utilized for engineering biological systems, incorporating novel functionality, and circumventing many of the complications associated with cells. The central dogma describes the information flow in biology consisting of transcription and translation steps to decode genetic information. Aminoacyl tRNA synthetases (AARSs) and tRNAs are key components involved in translation and thus protein synthesis. This review provides information on AARSs and tRNA biochemistry, their role in the translation process, summarizes progress in cell-free engineering of tRNAs and AARSs, and discusses prospects and challenges lying ahead in cell-free engineering.

## 1 Cell-Free Synthetic Biology

Cell-free reactions were first used in 1897 when Buchner showed that yeast extract can be used for the fermentation process (Buchner, 1897). In the last century when the scientific community was exploring the molecular biology of the cell and identifying the key components involved in the central dogma of life, cell-free studies were used as a tool to understand their fundamental biochemical functions and served as an additional tool to validate or support their hypothesis. A classic example is the requirement of template RNA for amino acid incorporation during protein synthesis which was proved *in vitro* by Nirenberg in 1961 using a cell-free system extracted from *E. coli* (Nirenberg and Matthaei, 1961). Cell-free biology can be defined as, “the reproduction, study, and exploitation of complex biological processes without intact cells” (Swartz, 2006). Research that was not possible with intact cells or constrained by the limitations or complexities of the cell, was made possible by cell-free systems. For example, lysate-based systems have been successfully used for the study and implementation of synthetic gene regulatory networks (Swank, Laohakunakorn, and Maerkl, 2019) and forward engineering of genetic oscillators (Niederholtmeyer et al., 2015). Cell-free systems also provided a faster and more convenient way to synthesize proteins using linear rather than circular DNA templates. Cell-free systems opened up the possibility to work with a range of organisms from conventional model organisms such as *E. coli* and yeast to more un-conventional systems such as *Bacillus megaterium* (Moore et al., 2018), *Clostridium autoethanogenum* (Krüger et al., 2020), and eukaryote-derived systems such as rabbit reticulocyte lysate (Gagoski et al., 2016).

Initially, cell-free systems were prepared using cell lysates, where cells were lysed, chromosomal DNA and cell membrane debris were removed, and the rest of the cellular contents were used for studies. Lysate-based systems suffered from batch-to-batch variation, hampering the ability to obtain consistent results (Hunter et al., 2018; Dopp, Jo, and Reuel, 2019). These systems also often contained inhibitory factors, nucleases, and proteases which lowered protein yield. Moreover, and in the context of molecular engineering of fundamental importance, lysates are complex and their composition is unknown.

More recently, a recombinant system called “protein synthesis using recombinant elements” (PURE) has been generated where all individual components required for transcription, translation, and energy regeneration are expressed, purified, and then reconstituted to create a cell-free system. PURE consists of 36 reconstituted proteins capable of cell-free transcription and translation. Proteins were purified using His-tag–based affinity chromatography (Shimizu et al., 2001). A recombinant-based cell-free system was made possible as a result of an improved understanding of cellular biochemistry and the molecular machinery involved in transcription and translation. Various studies on the PURE system were performed to make PURE preparation easier and to decrease its cost (Shepherd et al., 2017; Villarreal et al., 2018; Lavickova and Maerkl, 2019). Productivity and functionality was increased by adjusting the various PURE components (Li et al., 2014) and by supplementing additional factors to the system (Maddalena et al., 2016; Li et al., 2017b). With the advent of cell-free systems and the PURE system becoming more accessible and affordable, it is beginning to be used in various applications. The PURE system has been explored as a platform for producing therapeutic proteins (Cai et al., 2015; Dondapati et al., 2020) and for molecular diagnostics (Pardee et al., 2016). The modular nature of PURE has made this system also an appealing starting point for bottom-up synthetic cell approaches (Niederholtmeyer, Stepanova, and Maerkl, 2013; Lavickova, Laohakunakorn, and Maerkl, 2020). We have recently written a comprehensive review covering various aspects and applications of cell-free synthetic biology (Laohakunakorn et al., 2020), and the focus of this review lies on aminoacyl tRNA synthetase and tRNA biochemistry and engineering in the context of cell-free systems.

## 2 The Genetic Code

### 2.1 Discovery of the Genetic Code

Genetic information in biology is decoded through transcription and translation steps to result in RNA and proteins, respectively. Scientific activities in the last century helped us reach our current understanding of the steps involved in processing genetic material. In the following, a brief history of the discovery of key steps and components involved in the central dogma is discussed. In the early 20^th^ century, the scientific community believed that proteins should be the genetic material as they are structurally diverse being made from 20 different building blocks. However, nucleic acids have only 4 bases as their building blocks and were thus thought less likely to be the carrier of genetic information.

The nucleic acids (DNA and RNA) were first discovered in 1869 by Friedrich Miescher and were termed “nuclein” since they were found in the nucleus (Miescher, 1869). Many years after his discovery, Levene in 1919 identified the components of nucleic acid and the sugar group of nucleotides (Levene, 1919). Experiments by Avery and his colleagues in 1944 provided definitive proof that DNA is the genetic information with its transforming ability in bacteria (Avery, Macleod, and McCarty, 1944). On the other hand, proteins were conclusively proved not to be the genetic material by Hersley and Chase in 1952 (Hershey and Chase, 1952), and the work by Rosaling Franklin, Maurice Wilkins, James Watson, and Francis Crick leading to the 1953 paper describing the three-dimensional structure of the genetic material DNA (Watson and Crick, 1953), and its double-helical structure with base-pairing according to Chargaff’s rules (Chargaff, 1950).

The involvement of messenger RNA (mRNA) in the central dogma was floated since 1947 but the experimental discovery came in the year 1961 by Francois Gros and Francois Jacob (Jacob and Monod, 1961). For transfer RNA (tRNA), Crick hypothesized the existence of “adaptor” molecules, which are unstable and help carry the amino acids to the ribosomes in the cytoplasm for protein synthesis (Crick, 1955). tRNA was discovered by Paul Zamecnik in 1958 as a soluble RNA intermediate in protein synthesis and was the first non-coding RNA to be discovered (Hoagland et al., 1958). The ribosomal complex consists of ribosomal RNA (rRNA) and proteins, which is the site of protein synthesis located in the cytoplasm. It was discovered by George Palade in 1955 as small cytoplasmic bodies (Palade, 1955). Aminoacyl tRNA synthetases (AARSs) were first identified as activating enzymes in 1958, responsible for activating amino acids in the presence of ATP. Only after undergoing this activation step were amino acids able to participate in protein synthesis (Hoagland, Keller, and Zamecnik, 1956).

The genetic code linkage between nucleic acids and proteins was discovered by Marshall Nirenberg in 1961 (Nirenberg and Matthaei, 1961). Nirenberg’s work showed that codon triplets of RNA gave rise to amino acid sequences during protein synthesis. This work laid the foundation for establishing the codon–amino acid relationship in protein synthesis, which is referred to as the second genetic code.

### 2.2 Current Understanding of the Genetic Code

Many components are required to implement the genetic code and key components are DNA, DNA polymerase, RNA polymerase, mRNA, tRNA, ribosomal complex, amino acids, and AARS. Genetic code implementation is a result of specific interactions between these components. There is a nucleotide-based world (DNA and RNA) and an amino acid–based world (proteins). DNA replication duplicates and maintains the genetic code. The transcription step results in single-stranded RNA molecules using DNA as its template and the translation step uses RNA as its template to synthesize protein peptides and completes the decoding of genetic information. The translation process bridges the nucleotide world and the amino acid world. Specifically, AARS and tRNA connect the two worlds. Now we know that AARSs are the enzymes responsible for charging cognate amino acid onto its cognate tRNA. The fidelity of the translation process is hugely dependent on the specificity of the AARS enzymes. These enzymes have a direct influence on the protein synthesis process, segregating proteogenic amino acids from non-proteogenic amino acids. The next section contains details on tRNAs and AARSs focusing on synthesis, biochemistry, function, and editing activity.

## 3 Biochemistry of tRNA and Aminoacyl tRNA Synthetases

This section focuses on providing basic information on the structure, biochemistry, mode of action, classification, and the role of tRNA and AARS in the protein synthesis process. We are exclusively discussing *E. coli* AARSs and tRNAs unless otherwise indicated.

### 3.1 tRNA

**FIGURE 1:**
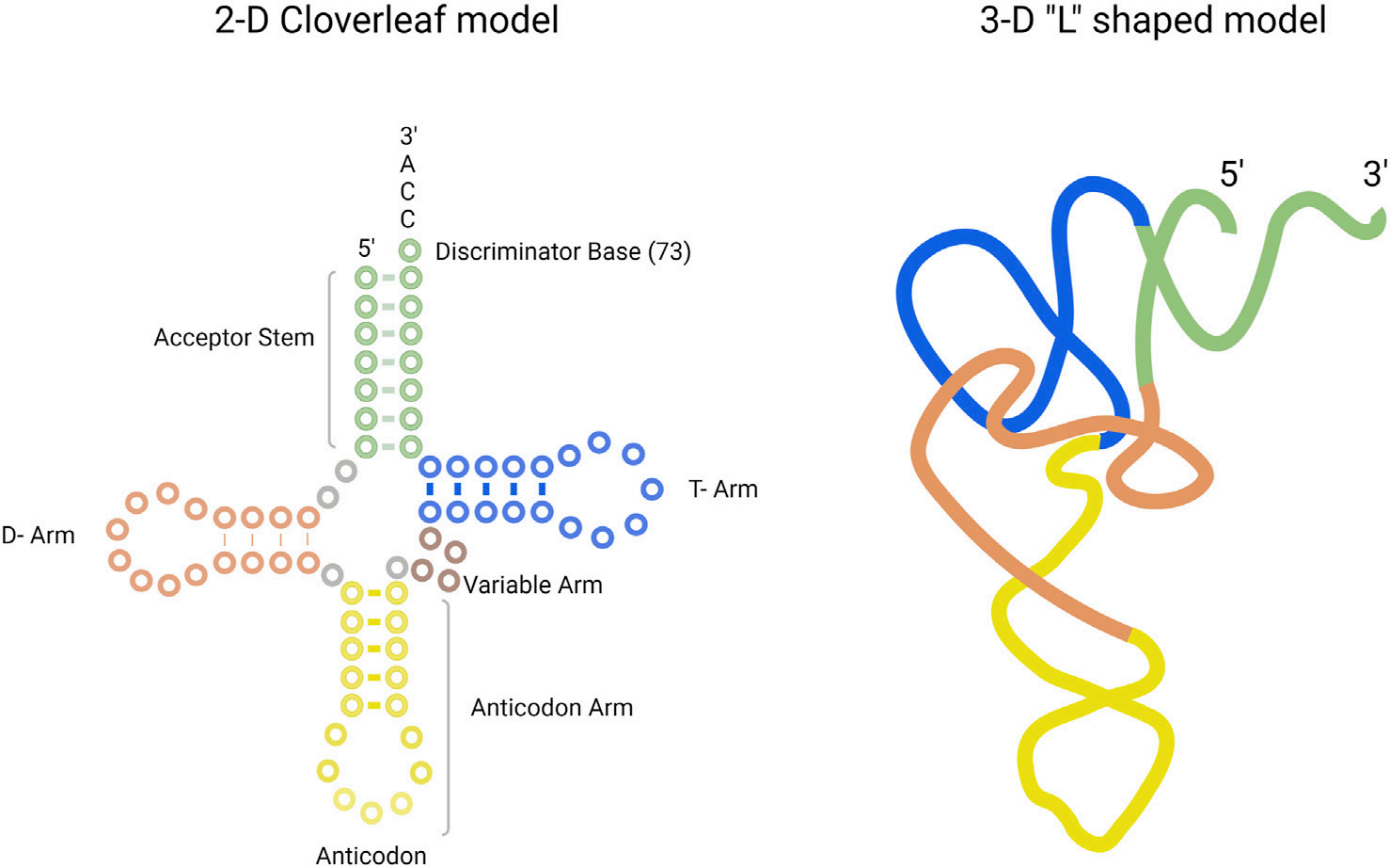
Structure of tRNA presented in the 2-D cloverleaf model (left) and in the 3-D “L” shaped model (right). The tRNA consists of acceptor stem (green), T-arm (blue), D-arm (orange), anticodon arm (yellow), and variable arm (purple).

The tRNA molecule is a single-stranded, non-coding RNA molecule. The general structure of tRNA in 2-D and in 3-D is provided in Figure 1. The tRNA structure consists of the following: the anticodon arm, D-arm, T-arm, acceptor stem, and the variable arm. Bases in the anticodon arm of tRNA molecules read the genetic information in mRNA codons and contain the corresponding amino acid present in the 3′-CCA sequence of the acceptor stem. tRNAs from the same species can exhibit a difference in sequence length, size of the variable arm, and length of acceptor stem. For example, tRNA^Sel^, the tRNA for selenocysteine amino acid in *E. coli*, is 95 nucleotides in length. On average, tRNA sequence length ranges from 75 to 90 nucleotides (Shepherd and Ibba, 2015).

Genes coding for tRNA are mostly arranged in groups in bacterial chromosomes with multiple copies present. Transcription is performed by RNA polymerase and results in tRNA precursor transcript having additional nucleotides on both 5′ and 3′ ends. Each tRNA transcript undergoes a maturation process where nucleotides are removed, specific nucleotide modifications occur, and structural integrity is gained, resulting in the cloverleaf shape. After maturation, a tRNA is available for amino acid charging at the 3′end by an AARS (Shepherd and Ibba, 2015).

The number of tRNAs present in an organism is dependent on codon usage. From a theoretical point of view, there are 64 (4^3^) different codon sequences available. However, only 61 different tRNAs are used, each corresponding to a particular codon, and the remaining three codons (UAA, UAG, and UGA) are called stop codons and do not have a corresponding tRNA. These 61 tRNAs are shared amongst 20 amino acids. The number of tRNA acceptors for each amino acid is not the same and varies across amino acids. For example, there exists only one tRNA for the amino acid methionine with codon AUG. On the other hand, multiple tRNAs can carry the same amino acid at their 3’ end and such tRNA groups are referred to as isoacceptors. For example, there are six isoacceptor tRNAs for the amino acid lysine with codons UUA, UUG, CUA, CUU, CUG, and CUC.

### 3.2 Aminoacyl tRNA Synthetases

**FIGURE 2:**
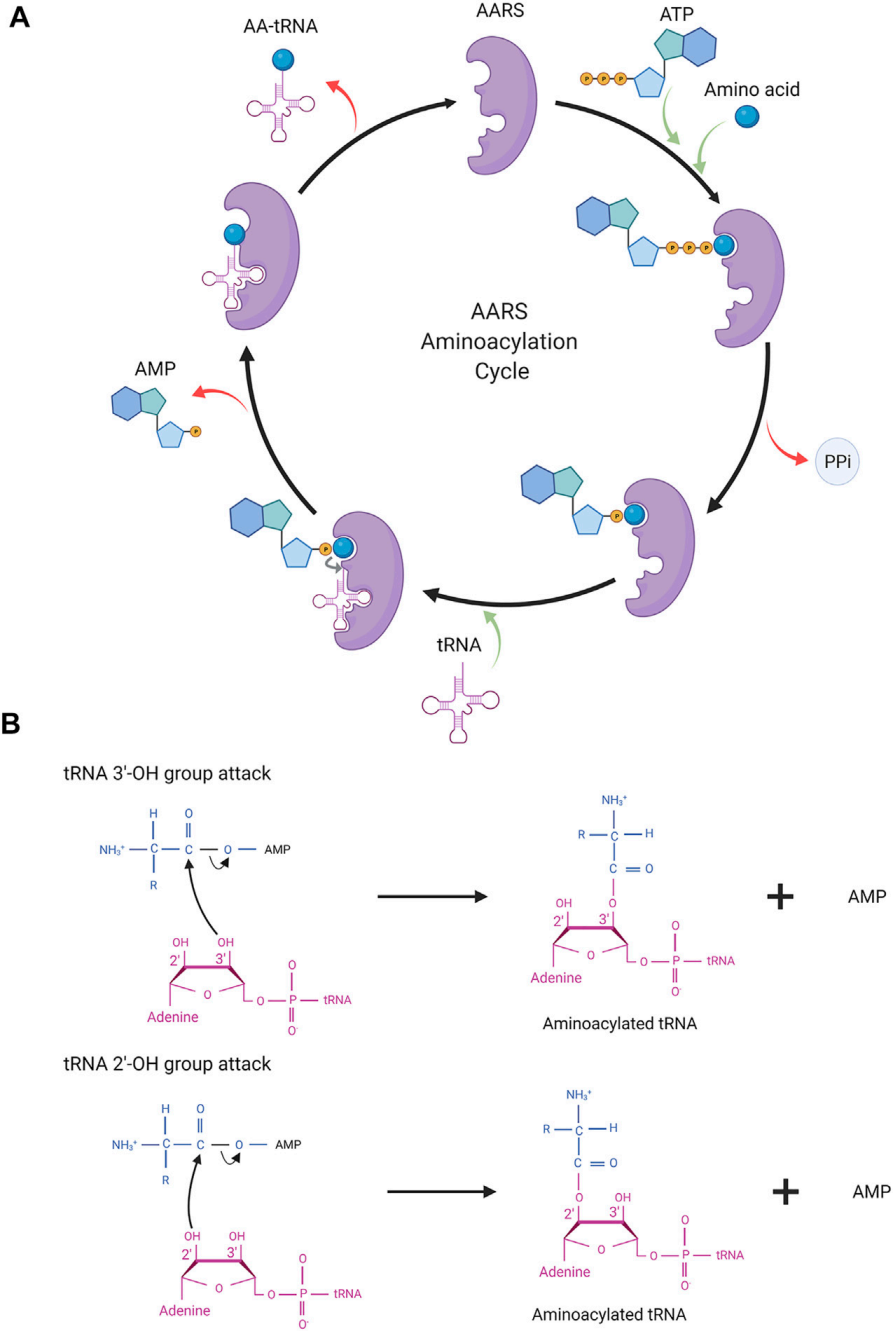
**(A)** AARS aminoacylation process. In the first step, AARS binds to the ATP and amino acid to form an aminoacyl intermediate. In the last step, the amino acid is transferred onto tRNA resulting in activated tRNA being ready for the translation process. AARS becomes free for the next cycle of aminoacylation. **(B)** Depicting the molecular structure of terminal adenosine of tRNA and the attack of the hydroxyl group (2′/3′) in AA-AMP intermediates.

AARSs are a family of enzymes responsible for adding an amino acid onto its cognate tRNA molecule. They are the enzyme implementing the genetic code. There are 21 AARS enzymes present, one for each amino acid except lysine, which has two AARSs. In addition to these 21 AARSs for proteogenic amino acids, there are AARSs for non-proteogenic amino acids such as pyrrolysyl tRNA synthetase and phosphoseryl tRNA synthetase. These additional AARSs are found in archaea and bacteria. Each tRNA has a particular AARS for its activation. In general, tRNA charging by AARSs with amino acid takes place in two steps (Figure 2).

In the first step, amino acid activation takes place. ATP and amino acid bind to the AARS enzyme triggering a nucleophilic attack of the amino acid carboxyl oxygen to the α-phosphate group of ATP. This results in amino acid adenylate intermediate (AA–AMP) and release of pyrophosphate (PPi). In the second step, one of the hydroxyl groups of adenosine (3′-OH/2′-OH) in tRNA attacks the carboxyl carbon of AA–AMP intermediate resulting in the transfer of amino acid to tRNA. The amino acids and tRNAs are linked by an ester bond. This step results in tRNA-AA, and AMP, which are released from the catalytic site of the enzyme and the AARS enzyme is free for the next cycle. In general, for the amino acid activation step, tRNA is not required but some AARSs such as GlnRS, GluRS, ArgRS, and class I LysRS require tRNA as a prerequisite for amino acid activation. Activated tRNA-AA binds with the elongation factor, EF-TU, and when reaching the ribosome participates in translation.

#### 3.2.1 Aminoacyl tRNA Synthetases Classes

The 23 AARS enzymes are classified into two classes depending on the structure of the active site. In class I AARSs, the active site contains the Rossmann fold with five parallel β sheets connected by α helices. The Rossmann fold contains highly conserved motifs: HIGH and KMSKS, and both motifs are connected by sequence stretches called CP1. Class II AARS active sites have several parallel β strands flanked by α helices. Class I enzymes are either monomeric or dimeric and class II enzymes are dimeric or tetrameric. There exist many differences between class I and class II AARSs. Amino acid charging takes place at the 3′-OH group of tRNA in class I AARSs and PheRS, and 2′-OH group in class II AARSs. ATP binding during amino acid activation differs in both classes. ATP binds in an extended conformation in class I AARSs and in a kink conformation in class II AARSs. AARSs differ in the way they bind tRNA with class I AARSs binding the minor groove of tRNA and class II AARSs binding the major groove. In terms of reaction catalysis, the rate-limiting steps between both classes differ as well. In class I AARSs, the release of activated tRNA (tRNA-AA) is the rate-limiting step whereas in class II AARSs it is the amino acid activation step. Each AARS class is further categorized into subclasses depending on the type of amino acid charged by the enzyme. Across both class I and class II, subclass A recognizes aliphatic and thiolated amino acids, subclass B recognizes charged polar amino acids, and subclass C recognizes aromatic amino acids (Rubio Gomez and Ibba, 2020).

#### 3.2.2 Substrate Recognition by Aminoacyl tRNA Synthetases

Amino acids are much smaller in size than tRNAs, and so fewer chemical moieties are available for AARSs to distinguish cognate from non-cognate amino acids. Generally, amino acids are recognized based on their size, functional group, and ability to bind with metal ions present in the active site of the enzyme. In the enzyme PheRS, a conserved alanine residue helps discriminate phenylalanine over tyrosine (Reynolds et al., 2010). Glycine, the smallest amino acid, is recognized by the high negative charge in the binding pocket of its AARS. Five different conserved negative charges are used to identify glycine. Two threonine residues in the GlyRS binding pocket help to prevent other amino acids from activating. Crystallographic studies have shown that zinc metal ions in the active site of the enzyme help distinguish cognate from non-cognate amino acids (Valencia-Sánchez et al., 2016). In the case of ThrRS (Sankaranarayanan et al., 2000) and CysRS (Zhang et al., 2003), the cognate amino acid is selected by its ability to interact with zinc ions present in the active site whereas non-cognate amino acids fail to do so.

**FIGURE 3:**
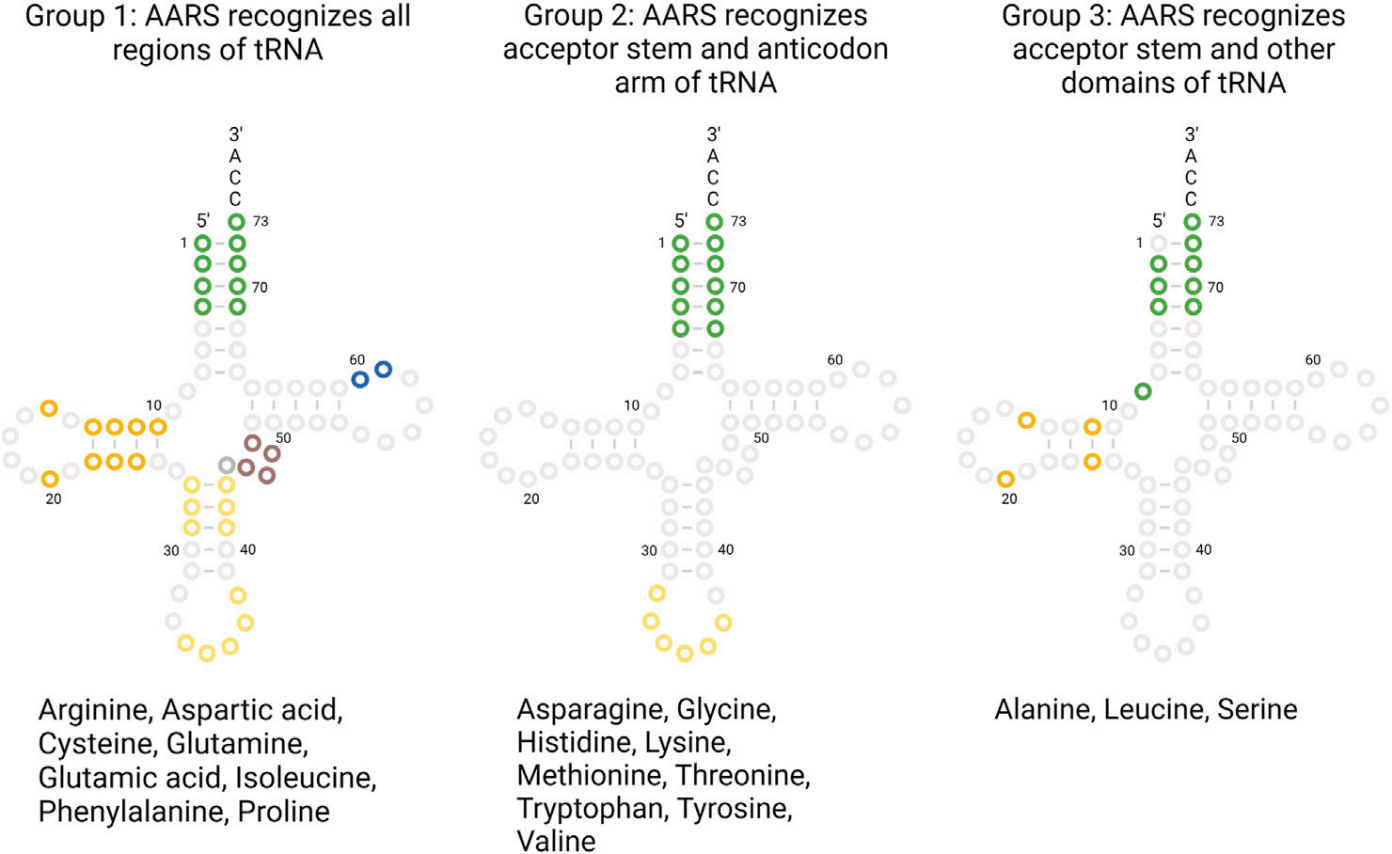
AARS identity elements on tRNA. AARSs are grouped based on the location of the identity elements present on tRNA regions. The list of AARS under each group and the tRNA bases utilized for recognition are highlighted.

Each AARS has a specific binding pocket for tRNA. Selecting the cognate tRNA is crucial for ensuring translation fidelity. Initial binding of tRNA to AARS is fast and non-specific and governed by electrostatic interaction. Upon initial binding, specific interactions between tRNA and AARS ensure recognition of the correct tRNA. Specific interactions are formed more slowly, accompanied by conformational changes in the active site of AARS. Specific interactions are mediated by identity elements such as modified nucleotides, conserved residues, base stacking, and different tRNA arm lengths. The identity elements include determinants and anti-determinants. Determinants favor binding of cognate tRNAs with AARSs while anti-determinants disfavor binding of non-cognate tRNAs. The most commonly used identity elements are the anticodon bases 34, 35, and 36 in the anticodon arm and the discriminatory base 73 in the acceptor stem of the tRNA. In the case of AlaRS, tRNA is recognized exclusively based on the presence of G3-U70 base pair (McClain and Foss, 1988a). When non-cognate tRNAs were engineered *in vitro* containing the aforementioned base pair, AlaRS recognized those tRNAs and charged them with alanine (Hou and Schimmel, 1988; McClain and Foss, 1988a). For SerRS, the length of the variable arm is more crucial for its discrimination than sequence (Park and Schimmel, 1988; Asahara et al., 1993). The complete list of identity elements and their location in the tRNA for each AARS from *E. coli* is provided in Table 1. All AARSs are classified into three groups based on the location of the tRNA identity elements. AARSs in group 1 have identity elements located in all regions of the tRNA, namely, the acceptor stem, anticodon arm, other domains (T-arm, D-arm, and the variable arm). AARSs in group 2 have identity elements located only in the acceptor stem and anticodon arm. AARSs in group 3 have identity elements located in the acceptor stem and other domains of the tRNA but not in the anticodon arm. The list of AARSs in each group and the location of their identity elements on tRNA are provided in Figure 3.

**TABLE 1:** List of tRNA identity elements and their location on tRNA for aminoacylation by AARS from *E. coli*. Identity elements for fMet are provided in italics and bold.

AARS	Identity element location				References
	Acceptor stem	Anticodon arm		Other domains (d-arm/T-arm/variable arm)	
		Anticodon	Other location		
Alanine	A73,			G20	Hou and Schimmel, 1988; McClain and Foss, 1988a; Francklyn et al., 1992
	G2:C71,				
	G3:U70,				
	G4:C69				
Arginine	A/G73	C35, U/G36		A20	McClain and Foss, 1988b; Schulman and Pelka, 1989; McClain et al., 1990; Tamura et al., 1992
Asparagine	G73	G34, U35, U36			Shimizu et al., 1992; Li et al., 1993
Aspartic acid	G73,	G34, U35, C36,	C38	G10	Hasegawa et al., 1989; Nameki et al., 1992
	G2:C71				
Cysteine	U73,	G34, C35, A36		G15: G48,	Pallanck et al., 1992; Shimizu et al., 1992; Hou et al., 1993; McClain, 1993; Komatsoulis and Abelson, 1993; Hamann and Hou, 1997
	G2:C71,			A13:A22	
	C3:G70				
Glutamine	G73,	C/T34, U35, G36	A37, U38	G10	Rogers and Söll, 1988; Jahn et al., 1991; Hayase et al., 1992; Ibba et al., 1996
	U1:A72,				
	G2:C71,				
	G3:C70				
Glutamic acid	G1:C72,	U34, U35,	A37	U11:A24, U13:G22-A46, 47	Normanly et al., 1990; Sylvers et al., 1993; Gregory and Dahlberg, 1995; Sekine et al., 1996
	U2:A71				
Glycine	U73,	C35, C36			Shimizu et al., 1992; Francklyn et al., 1992; McClain et al., 1991
	G1:C72,				
	C2:G71,				
	G3:C70				
Histidine	C73,	Anticodon			Shimizu et al., 1992; Francklyn et al., 1992; Himeno et al., 1989; Francklyn and Schimmel, 1990; Yan and Francklyn, 1994; Yan et al., 1996
	G1				
Isoleucine	A73,	G34, A35, U36	A37, A38	U12:A23, C29:G41	Pallanck and Schulman, 1991; Muramatsu et al., 1988; Nureki et al., 1993; Nureki et al., 1994
	C4:G				
	69				
Leucine	A73			U8:A14	Normanly et al., 1992; Asahara et al., 1993
Lysine	A73	U34, U35, U36			Normanly et al., 1990; McClain et al., 1990; Tamura et al., 1992
Methionine ***fmet***	A73,	C34, A35, U36	***C32, U33, A37***		Uemura et al., 1982; Schulman and Pelka, 1988; Meinnel et al., 1993; Lee et al., 1992
	U4:A69,				
	A5:U68				
	***G2:C71,***				
	***C3:G70***				
Phenylalanine	A73	G34, A35, A36	G27:C43,	U20, G44, U45, U59, U60	Pallanck and Schulman, 1991; McClain and Foss, 1988c; Peterson et al., 1993; Peterson and Uhlenbeck, 1992
			G28:C42		
Proline	A73, G72	G35, G36		G15:C48	Shimizu et al., 1992; McClain et al., 1994; Liu et al., 1995
Serine	G73,			C11:G24 (variable arm)	Normanly et al., 1992; Himeno et al., 1990; Rogers and Söll, 1988; Normanly et al., 1986; Sampson and Saks, 1993; Asahara et al., 1994; Saks and Sampson, 1996
	C72,				
	G2:C71,				
	A3:U70,				
	C11:G24, G/A4:T/C69				
Threonine	G1:C72,	G34, G35, U36			Schulman and Pelka, 1990; Hasegawa et al., 1992
	C2:G71				
Tryptophan	G73,	C34, C35, A36			Himeno et al., 1991; Pak et al., 1992; Rogers et al., 1992
	A1:U72,				
	G2:C71,				
	G3:C70				
Tyrosine	A73	U35			Hou and Schimmel, 1989; Bedouelle, 1990; Himeno et al., 1990; Sherman et al., 1992
Valine	A73,	A35, C36			Chu and Horowitz, 1991; Tamura et al., 1991; Pallanck and Schulman, 1991
	G3:C70,				
	U4:A69				

Additional discrimination comes from the kinetic aspect of binding to discriminate cognate tRNAs. Aminoacylation with cognate tRNAs is more influenced by K_cat_ values than K_M_ values (Ebel et al., 1973). Evolutionary conservation of the identity elements in tRNAs suggests their importance, even though these elements do not directly contribute to protein synthesis.

#### 3.2.3 Proofreading Mechanism by Aminoacyl tRNA Synthetases

Pauling in 1958, theoretically predicted that amino acid misincorporation during translation should be about 1 in 200 (Pauling, 1958). However, *in vivo* experiments showed that this error rate is about 1 in 3,000 (Loftfield, and Vanderjagt, 1972). Aminoacylation by AARS has an error rate of about 1 in 10,000. This led to the suggestion of some editing mechanism being in place to account for these observations. The low error rate for AARSs is due to better recognition of cognate substrates and a proofreading/editing mechanism. This section briefly describes the editing/proofreading mechanism used by AARSs to ensure faithful aminoacylation.

Fersht in 1977 proposed a “double sieve model” to explain the low error rate and presence of separate catalytic active and editing sites. According to this model, the active site of the enzyme acts as a first “coarse” sieve to filter out amino acids that are larger than the cognate amino acid. Amino acids which are similar to or smaller than cognate still will become activated in the active site. The second “fine” sieve is the editing site capable of hydrolysis, which has a pocket size smaller than the cognate amino acid. The editing site serves to de-acylate any of the mischarged smaller amino acids which passed through the first sieve (Fersht, 1977). This way, de-acylation of cognate amino acid is prevented as it cannot enter the editing site. Evidence for the presence of a separate editing site is seen in 10 AARS from both classes I and II.

Editing activity can be divided into pre-transfer editing and post-transfer editing. In pre-transfer editing, editing occurs before the amino acid is transferred to tRNA, and in post-transfer editing, editing occurs after the amino acid is transferred to tRNA. Most AARSs use one of these editing mechanisms, but some AARS such as LeuRS and ValRS use both mechanisms.

Pre-transfer editing occurs after the formation of amino acid adenylate (AA–AMP) but before transfer to tRNA. Pre-transfer editing is seen in both AARS classes. Pre-transfer editing can occur in two methods. In the first method, AA–AMP is released by the enzyme to the cytosol and the phosphoester bond is spontaneously hydrolyzed. In the second method, enzymatic hydrolysis of AA–AMP occurs either in the active site or in a separate editing site. For example, thiolated non-proteogenic amino acids such as homocysteine and ornithine are cleared by pre-transfer editing in the active site of the enzyme by MetRS and LysRS.

Post-transfer editing occurs after the transfer of amino acid to tRNA and occurs in a separate editing site. This editing involves cleaving the ester bond between amino acid and tRNA. In general, once tRNA-AA is formed, amino acid triggers a conformational change in the 3′ end of tRNA and results in tRNA translocation. Translocation results in an amino acid being in the editing site where it is hydrolyzed. For class II AARS, mischarged tRNA is rapidly released and in those cases, enzymes are capable of recapturing these mischarged tRNA for editing. In the case of PheRS, PheRS competes for Tyr-tRNA^Phe^ with EF-TU to recapture and edit the tRNA.

The preference for the editing mechanism used is dependent on the rate of amino adenylate hydrolysis and transfer to tRNA. In case of a faster transfer rate to tRNA as in ValRS, post-transfer is preferred. For IleRS, both reaction rates are fairly equal and hence both editing mechanisms are used.

**FIGURE 4:**
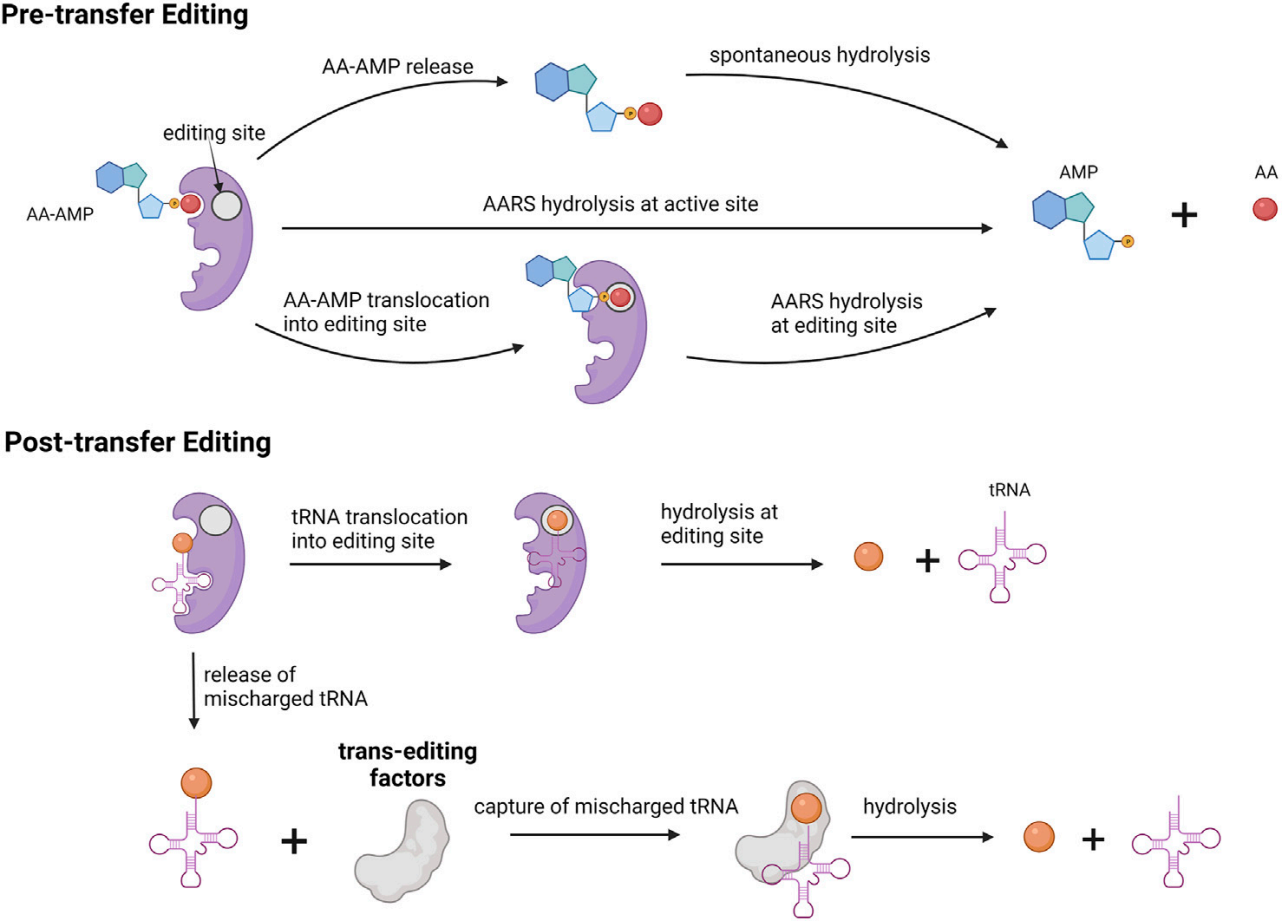
Editing mechanism for aminoacylation process. Pre-transfer editing occurs before amino acid gets charged onto tRNA. Post-transfer editing occurs after amino acid is charged onto tRNA. Trans editing factors are independent editing proteins involved in hydrolysis of mischarged tRNA.

There also exists a separate group of editing proteins that act independently of AARSs called trans-editing factors. These enzymes provide additional quality control in the editing process. The role of the trans-editing factors is to clear mischarged tRNAs before they reach the ribosome. D-aminoacyl-tRNA deacetylases are another class of trans-editing factors targeting in particular tRNAs charged with D-amino acids. The presence of such multiple editing mechanisms signifies the importance of the aminoacylation process and fidelity of the translation process. A schematic representation of various editing mechanisms for aminoacylation is provided in Figure 4.

#### 3.2.4 Aminoacylation Kinetics

The accuracy of protein synthesis relies on an AARS's ability to recognize cognate amino acids and tRNAs. Kinetic analysis is useful to develop the mechanism of action in the steps involved in aminoacylation. This section provides information about different kinetic approaches used to study tRNA aminoacylation.

The parameters widely used to describe the kinetics of AARSs are substrate affinity K_M_, enzyme turnover K_cat_, maximum velocity V_max_, and enzyme specificity K_cat_/K_M_. K_M_ refers to affinity of the enzyme to the substrate. K_cat_ is the catalytic constant for substrate to product conversion. K_cat_/K_M_ is the specificity constant or catalytic efficiency of the enzyme. Steady-state kinetics is useful for initial characterization of the enzyme and to measure kinetic parameters. Since steady-state kinetics are used generally, parameters obtained can be compared across systems. For example, enzyme specificity for cognate and non-cognate amino acids can be measured and compared across AARSs. Steady-state measurements are usually performed with substrate concentrations much higher than enzyme since the assay follows product formation. Minimal material requirements and fast readout make the steady-state approach suitable for initial characterization. The drawback of steady-state kinetics is that elementary reactions cannot be characterized. To determine the rate of the aminoacylation process, ATP pyrophosphate exchange assays and aminoacylation assays are performed under steady-state kinetics.

The ATP–PPi exchange assay is based on the amino acid activation step. ATP and amino acids form aminoacyl adenylate intermediate (AA–AMP) with the release of pyrophosphate (PPi). In one approach, [^32^P]-PPi is used for the reaction. The radioactive group reacts with AA–AMP resulting in [^32^P]-ATP. This assay measures the exchange of [^32^P]-PPi into ATP to provide rate of the activation step. In another approach, radioactive [^32^P]-ATP is used for amino acid activation and the rate of ATP consumption is measured using activated charcoal or thin layer chromatography (TLC) plates.

Aminoacylation assays are dependent on the second step of amino acid transfer to tRNA. Amino acids radiolabeled with [^3^H] or [^14^C] are used to measure the rate of product AA-tRNA^AA^ formation over time. One drawback of using radiolabeled amino acids is that attaining saturating conditions is difficult. This can be challenging while determining the K_M_ value of tRNA for AARS. The aforementioned limitation can be overcome by using a radiolabeled [^32^P] group at the 3′ end of tRNA and unlabeled amino acids. In such a way, saturating amino acid concentration can be used in the assay. Table 2 contains the K_M_ and K_cat_ of amino acids for all AARSs from *E. coli* unless mentioned otherwise.

**TABLE 2:** K_M_ and K_cat_ values of amino acids for AARS from *E. coli* unless mentioned otherwise. Unit for K_cat_ is s^−1^ unless mentioned otherwise.

AARS	Amino acid		References
	K_M_ (µm)	K_cat_ (s^−1^)	
AlaRS	240 ± 50	33 ± 7	Hill and Schimmel, 1989
ArgRS	12	2.2	Lin et al., 1988; Airas, 2006
AsnRS	32	1.6	Madern et al., 1992
AspRS	60	18	Martin et al., 1997
CysRS	0.4 ± 0.1	680 ± 60 (nmol min^−1^ mg protein^−1^)	Komatsoulis and Abelson, 1993
GlnRS	0.114 ± 0.012	157 ± 7 min^−1^	Liu et al., 1998
GluRS	5	5.5 ± 1.0	Lapointe and Söll, 1972; Campanacci et al., 2004
GlyRS	0.03	0.31 ± 0.02 (*Homo sapiens*)	Ostrem and Berg, 1974; Cader et al., 2007
HisRS	1.4 ± 0.6	2.6 ± 0.4	Augustine and Francklyn, 1997
IleRS	2.1 ± 0.2	3.1 ± 0.2	Xu et al., 1994
LeuRS	15	3	Chen et al., 2000
LysRS	230 ± 20	0.34 ± 0.009	Wang et al., 2006
MetRS	1.2 ± 0.2	3.2 ± 0.2	Ghosh et al., 1991
PheRS	1.8 ± 0.2	65 ± 3 min^−1^	Moor et al., 2011
ProRS	250 ± 35	70 ± 25	Beuning and Musier-Forsyth, 2001
SerRS	0.56 ± 0.15	2.6 ± 0.4	Borel et al., 1994
ThrRS	12	0.3	Hirsh, 1968
TrpRS	0.53 ± 0.08	1.34 ± 0.26	Chan and Koeppe, 1994
TyrRS	3.3 ± 0.8	0.74 ± 0.06	Hamano-Takaku et al., 2000
ValRS	4.3	13.9	Tardif and Horowitz, 2004

Pre-steady state kinetics is used to study elementary reaction steps. The pre-steady state kinetic approach is used to study fast reactions, in the order of a few milliseconds, present at an early stage of the interaction. This approach is best for understanding the mechanistic action of interaction. Parameters like individual rate constants of the reactants can be determined using pre-steady state kinetics. For example, substrate-binding order in the active site, formation, and consumption of intermediates can be studied by pre-steady state kinetics. Rapid chemical quench and stopped-flow fluorimetry are generally used to study AARSs. Rapid kinetic approaches were used to mechanistically distinguish the two classes of AARSs. As mentioned earlier, in class I AARSs, product release of AA-tRNA^aa^ is the rate-limiting step, and in class II AARSs, amino acid activation is the rate-limiting step.

Rapid chemical quench is a discontinuous assay providing a direct readout of the rate of the radiolabeled product formed. Stopped-flow fluorimetry is a continuous assay and provides an indirect readout of reaction progress. Progress is dependent on changes in intrinsic tryptophan fluorescence correlated to reaction chemistry.

Table 3 contains the half-life of activated tRNA-AA measured in *E. coli* (Hentzen, Mandel, and Garel, 1972). The value represents the spontaneous hydrolysis rate of tRNA-AA at neutral or alkaline pH in a high ionic condition at 37°C. Under the same conditions, the stability of the ester bond depends purely on the amino acid attached to tRNA. Half-life for all amino acids but tryptophan is presented and ranges from 2 to 65 min.

**TABLE 3:** Half-life values of tRNA-AA from *E. coli*. Values obtained based on ester bond hydrolysis under neutral or alkaline pH in a high ionic medium at 37°C (Hentzen, Mandel, and Garel, 1972).

tRNA-AA	t _1/2_ (min)
Ala	6
Arg	12
Asn	11
Asp	11
Cys	16
Gln	9
Glu	9
Gly	8
His	16
Ile	65
Leu	7
Lys	14
Met	12
Phe	16
Pro	2
Ser	17
Thr	38
Trp	-
Tyr	15
Val	60

## 4 Applications of tRNA and Aminoacyl tRNA Synthetases in Cell-Free Systems

### 4.1 tRNA and Aminoacyl tRNA Synthetases *In Vitro* Synthesis

#### 4.1.1 tRNA Synthesis

The ability to synthesize tRNA *in vitro* was demonstrated in 1973 when T4 DNA was transcribed to yield T4 tRNAs when incubated with *E. coli* extract obtained after infecting with T4 bacteriophage (Nierlich et al., 1973). Currently, a more sophisticated one-pot method for *in vitro* tRNA synthesis has been developed (Korencić, Söll, and Ambrogelly, 2002). In this method, T7 RNAP is used for tRNA transcription from a ds/ssDNA hybrid template. Transcribed tRNAs were shown to be produced in full length and aminoacylated by AARSs. This method did not have as high a yield as the control method using plasmid DNA as a template, but is a useful approach to produce functional tRNAs *in vitro* (Korencić, Söll, and Ambrogelly, 2002). *In vitro* synthesis of functional tRNA has made it easier to study interactions of tRNA with other components such as mRNA (Fauzi, Jack, and Hines, 2005) and AARSs (Wang et al., 2015), to investigate tRNA stability *in vitro* (Serebrov et al., 1998), and to determine mutational effects on tRNA function (Tamura et al., 1992).

The ability of *in vitro* transcribed tRNA to decode codons on mRNA during translation was studied as well. In one study, native tRNAs were depleted from the cell lysate and replaced with an *in vitro* transcribed tRNA subset. The cell lysate containing replenished tRNA was found to produce proteins and was able to decode all 61 codons with 48 transcribed tRNAs (Cui et al., 2015). This way, the tRNA pool directing the translation process can in principle be fully customized. In a similar study, efforts were taken to identify the minimal number of tRNAs required to support protein translation. The minimal set of tRNAs required for translation was explored by generating an entire set of 21 tRNAs *in vitro*, and they were shown to be able to synthesize proteins. A reduced number of tRNA was possible by using a single codon for each amino acid. The tRNAs were chosen such that they did not require modifications after synthesis. Furthermore, *in vitro* transcribed tRNAs provide flexibility in redesigning the genetic code and facilitate site-specific incorporation of amino acids. Protein yield obtained using this reduced set of tRNA was shown to be up to 40% compared to the native system (Hibi et al., 2020).

#### 4.1.2 Aminoacyl tRNA Synthetases Synthesis

From a synthetic biology perspective, the ability to express and sustain proteins is crucial for building a self-replicating cell. To this end, AARS proteins were expressed individually in the PURE system, and it was demonstrated that all AARSs except PheRS were expressed as soluble proteins (Awai, Ichihashi, and Yomo, 2015). These expressed proteins were functional with activity on par with their native counterparts purified from *E. coli*. The reason for the inactivity of PheRS was associated with insufficient formation of active dimers since dimer formation requires longer incubation at 4°C and low salt concentrations *in vitro* (Awai, Ichihashi, and Yomo, 2015). In a similar attempt, all 20 AARSs were expressed using a polycistronic plasmid encoding 32 proteins in total using the PURE system. The PURE system was able to synthesize all 32 proteins, including all 20 AARSs as confirmed by mass spectrometric analysis but functionality was not assessed (Doerr et al., 2021). Recently, in the bid to construct a self-replicating synthetic cell, a modified PURE system was used to demonstrate self-regeneration of up to 7 AARSs in a microfluidic reactor for more than 24 h (Lavickova, Laohakunakorn, and Maerkl, 2020).

### 4.2 tRNA and Aminoacyl tRNA Synthetase Engineering

#### 4.2.1 tRNA Engineering

Major work on tRNA engineering was achieved *in vivo* in regard to genetic code expansion (GCE). GCE involves the increase in the genetic code alphabet by introducing new base nucleotides (Hoshika et al., 2019), unnatural base pairs (UBPs) (Mukba et al., 2020), creating new codons, and increasing the codon size to 4 (quadruplet codon) (Hohsaka et al., 2001). The aforementioned approaches were used for incorporating modified or non-canonical amino acids (NC-AAs). Another approach is to reassign existing codons for NC-AA incorporation. Incorporating a NC-AA requires an orthogonal translation system (OTS) with a tRNA/AARS pair that does not cross-react with the endogenous tRNA/AARS present in the system. There should not be any interference of the endogenous AARS with the exogenous AARS in its ability to recognize the exogenous tRNAs and NC-AAs, and vise-versa. Orthogonality can be achieved by utilizing tRNA/AARS pairs from phylogenetically distinct species, and this approach takes advantage of differences in codon usage. Another approach takes advantage of the difference in tRNA recognition by AARSs (Doctor and Mudd, 1963). Orthogonal pairs TyrRS/tRNA-Tyr obtained from archaea *Methanococcus jannaschii* and pyrrolysyl-(Pyl)RS/tRNA-Pyl from *Methanosarcina barkeri* are most commonly used for incorporating NC-AAs. The open nature of cell-free protein synthesis (CFPS) provides a higher degree of freedom for structurally and functionally diverse NC-AAs to be incorporated. Parameters like cellular toxicity, viability, and cross membrane transport are not constraints for CFPS allowing incorporation of many different NC-AAs.

Depending on the application, either site-specific or residue-specific incorporation is used. In site-specific incorporation, a NC-AA is incorporated into a specific location whereas in residue-specific incorporation a NC-AA is incorporated into all sites encoded by a specific codon. In site-specific incorporation, both sense codons and nonsense codons were utilized for NC-AA incorporation. Modifications are introduced in the identity elements present in the tRNA, mostly in the anti-codon arm and in the acceptor stem, to favor NC-AA incorporation. While using a nonsense codon, a stop codon (UAG, UGA, and UAA) is reassigned to incorporate NC-AA instead. The amber codon (UAG) is usually used since this codon is least used as a termination signal in *E. coli*. In the input DNA sequence, all instances of the amber codon are changed to either one of the other two stop codons which frees the amber codon for reassignment to a NC-AA. The stop codon UAG is then reassigned to the tRNA containing NC-AAs and the tRNA anticodon arm is modified to CUA. With this approach, NC-AAs can be site-specifically incorporated at multiple places (Hong et al., 2014; Martin et al., 2018). Similarly, tRNA suppressor targeted toward opal (UGA) and ochre (UAA) codons were developed and utilized for *in vitro* NC-AA incorporation (Gubbens et al., 2010). As for sense codons, amino acids such as leucine and arginine have up to 6 codons, therefore; 6 tRNAs code for the same amino acid. One of the least commonly used isoacceptor tRNAs is generally used for codon reassignment. A report from 2016 demonstrated site-specific incorporation using sense codon reassignment using *in vitro* transcribed tRNAs charged with NC-AA in the PURE system. One of the codons for valine (GUG), arginine (CGC), and glycine (GGC) was reassigned to a different NC-AA (Iwane et al., 2016). This approach suffered from low efficiency in NC-AA incorporation due to wobble decoding, the ability of the tRNA to recognize more than one codon. The commercially available FluoTech system has fluorescently modified lysine instead of lysine. The codon AAA for lysine is reassigned to BODIPY lysine. Here, tRNA pre-charged with BODIPY lysine is added to the *in vitro* protein expression system, incorporating fluorescent lysine into proteins. It should be noted that the native tRNA_AAA-lys_ is still present in the system, leading to partial residue-specific incorporation of the fluorescent lysine. This system is used for easy detection of *in vitro* protein expression. For residue-specific incorporation, all of the sense codons for a particular amino acid are reassigned to the NC-AA. The native amino acid is replaced by a NC-AA in the amino acid pool. All tRNAs now carry the NC-AAs. Using an *E. coli* cell-free system canavanine amino acid, a toxic analog of arginine, was incorporated into the model protein at all locations of arginine (Worst et al., 2015).

As an alternative approach for NC-AA incorporation, quadruplet codons were used for tRNAs carrying the modified amino acids. The number of anticodons present in the anticodon arm is increased to 4 (UCCA) to match the corresponding 4-letter codon (AGGU). The modified amino acid was shown to be incorporated at multiple instances and up to two distinct NC-AA were incorporated with this approach. A modified nitrophenylalanine-tRNA was used to decode the 4-letter codons. Such a modified tRNA allowed incorporating NC-AAs with better efficiency in an *E. coli in vitro* translation system (Hohsaka et al., 2001). So far, tRNAs were engineered to incorporate NC-AAs instead of a stop codon or amino acid (*via* sense codon). In an attempt to increase the amino acid diversity to more than 20, the codon table was split for amino acids arginine, glycine, and valine and, the free codons were assigned to three distinct NC-AAs, thereby increasing the total number of amino acids to 23, in addition to the 20 native amino acids (Iwane et al., 2016).

Charging of orthogonal tRNA with NC-AAs is a key step in the translation process and it is usually mediated by AARS enzymes. In addition to AARS enzymes, there are other methods such as the chemoenzymatic method, chemical method, and ribozyme-based approaches for acylation to charge tRNA with NC-AAs. These synthetic methods are utilized *in vitro* to generate pre-charged tRNAs with NC-AAs and can be directly supplemented into the cell-free system. Such pre-charged tRNAs are useful to learn more about single turnover translation. The key advantage of chemical acylation methods is that structurally and chemically diverse groups can be added onto tRNAs without the need to re-engineer AARS.

#### 4.2.2 Aminoacyl tRNA Synthetases Engineering

The central theme for AARS engineering is in genetic code expansion and incorporation of unnatural or NC-AAs during peptide synthesis. AARSs can be engineered by modifying the amino acid binding pocket, tRNA binding pocket, and editing domain for acylating NC-AAs onto tRNAs. In a relatively simple approach, native AARSs are used to incorporate unnatural amino acids. Here, the lack of specificity in substrate recognition by native AARSs was exploited to charge tRNAs with unnatural amino acids to participate in protein synthesis. For example, research in non-ribosomal peptides utilized this approach to demonstrate simultaneous incorporation of 10 different amino acid analogs using the PURE-based recombinant system. The amino acid analogs used were substrates for 12 native AARSs from *E. coli* (Josephson, Hartman, and Szostak, 2005). Even though it is simple, this approach does suffer from low efficiency and therefore yield. Moreover, if cognate amino acids are present, they will compete with the same codon and lower the incorporation of NC-AAs. In such cases, the relative ratio of NC-AA to cognate amino acid should be tightly controlled to favor NC-AA incorporation.

The second approach uses engineered AARSs with altered specificity for amino acids or tRNAs. In the report mentioned earlier, AARS substrate diversity was further expanded by a mutation in the binding pocket domain and editing domain to accept previously unaccepted amino acids. For example, in PheRS, a specific mutation at binding pocket domain Ala294Gly can accept the phenylalanine analog p-iodo-Phe. Similarly, inactivating the editing domain of LeuRS by Asp345Ala accepted allylglycine (Josephson, Hartman, and Szostak, 2005). Furthermore, studies have systematically explored the diversity of NC-AAs as substrates for incorporation in native and engineered AARSs. It affirms the polyspecificity of AARS in substrate selection and expands substrate diversity for engineering proteins with novel functional groups (Hartman, Josephson, and Szostak, 2006; Fan et al., 2014).

Cell-free systems opened up the use of insoluble, non-canonical AARS for NC-AA incorporation. Pyrrolysyl synthetase (PylRS) is one such AARS which had limited use due to difficulties in purification. PylRS and its mutants are utilized in more than 100 unnatural amino acid incorporations (Wan, Tharp, and Liu, 2014) and are highly useful due to their minimal cross-reactivity with the *E. coli* system. The expression of PylRS/tRNA pair from *Methanosarcina mazei* in *E. coli* helped obtain a cell-free extract containing the insoluble PylRS. This cell-free extract was used to demonstrate the site-specific incorporation of two analogs of pyrrolysine into a reporter gene (Chemla et al., 2015). However site-specific incorporation requires a faster and more accurate aminoacylation reaction between NC-AA and tRNA, there is a need for a better strategy to identify mutants with better incorporation efficiency.

Identification of correct mutants of orthogonal AARS for NC-AA incorporation is largely benefited from methods such as multiplex automated genome engineering (MAGE) and phage-assisted continuous evolution (PACE). MAGE is an *in vivo* continuous platform for large-scale introduction of allelic replacement in chromosomal DNA achieved by inactivation of the mismatch repair system by the *mutS* gene and λ-Red recombinase systems containing exo, beta, and gam proteins. ssDNA or oligo nucleotides (90 bases) are delivered to cells using electroporation and are introduced at the lagging strand of the replication fork at target locations. Each MAGE cycle requires about 2–2.5 h and higher diversity is generated at multiple locations by increasing the number of MAGE cycles as required. The process facilitates accumulation of a large number of mutations and increases the possibility of producing a beneficial protein phenotype (Wang et al., 2009). PACE is an autonomous directed evolution approach, where a modified M13 bacteriophage contains the gene of interest and is subjected to continuous cycles of mutagenesis and selection. A large number of mutations are continuously generated and selected (Esvelt, Carlson, and Liu, 2011). These methods helped generate and screen a large library of AARS mutants in a short period, thereby saving time and effort. Despite the aforementioned methods being developed *in vivo*, orthogonal AARSs can also be used in *in vitro* systems for high efficiency NC-AA incorporation (Amiram et al., 2015; Bryson et al., 2017). Similarly, computational techniques have been used to generate and screen AARS mutant libraries specifically targeted for ortho-nitrobenzyl tyrosine, an analog of tyrosine. The advent of such methods will pave the way for better and faster engineering of novel AARSs (Baumann et al., 2019).

Furthermore, cell-free systems are advantageous to study non-canonical functions of AARS in addition to tRNA charging. For instance, it is well established that AARSs are also involved in mRNA binding and regulation in yeast (Levi and Arava, 2019), and AARSs can autoregulate their own expression by binding to specific DNA in bacteria (Putney and Schimmel, 1981). Cell-free systems can be used to distinguish between canonical and non-canonical functions of AARSs. Readers interested in non-canonical functions of AARSs can refer to other comprehensive reviews (Ivanov, Moor and Lavrik, 2000; Guo and Schimmel, 2013).

##### 4.2.2.1 Aminoacylation Methods

**FIGURE 5:**
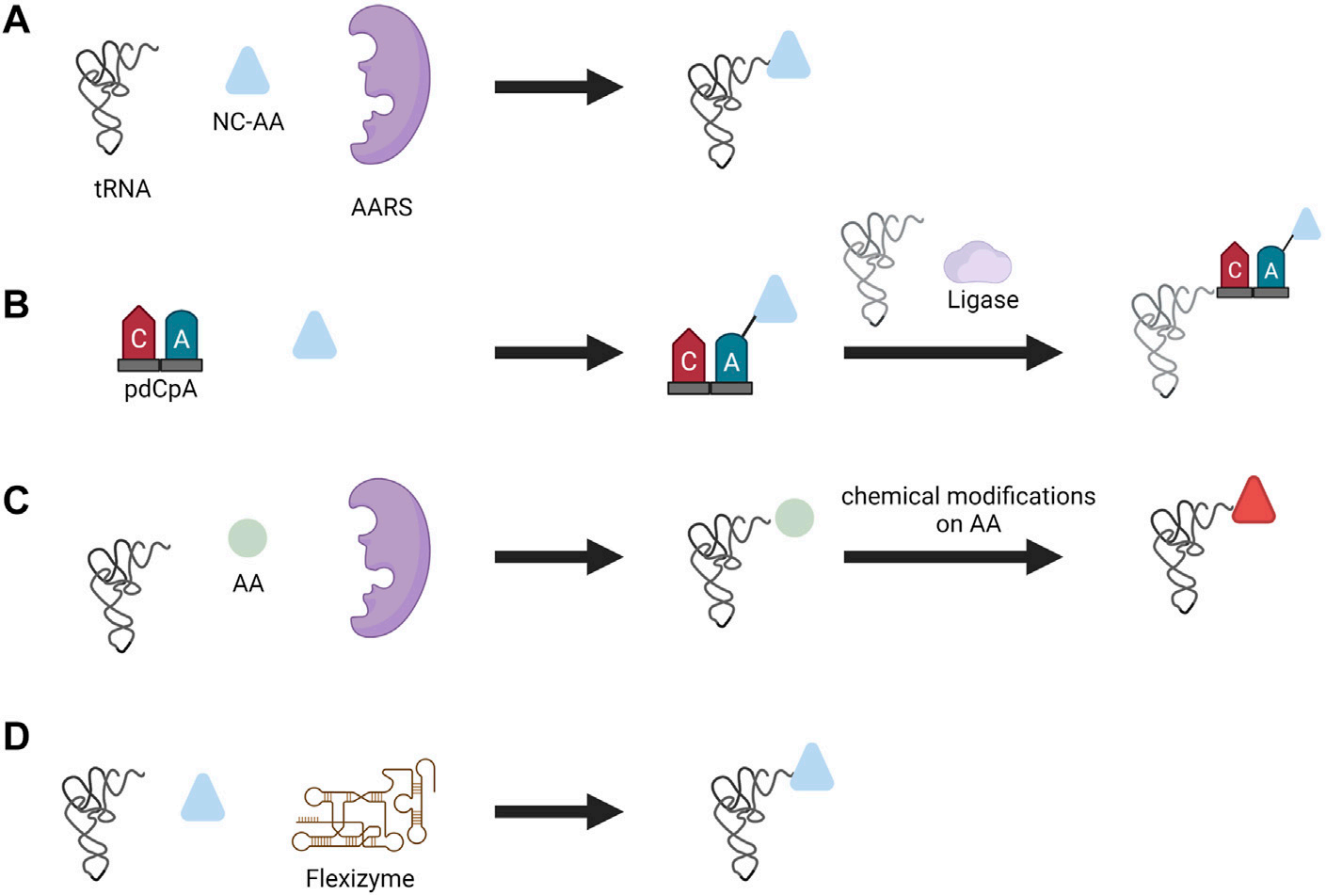
Different aminoacylation methods used for NC-AA incorporation. **(A)** Enzymatic aminoacylation by AARS, **(B)** chemoenzymatic method, **(C)** chemical method, and **(D)** flexizyme method.

AARS enzymatic reactions are not the only way to charge tRNAs with a NC-AAs. Other methods to charge tRNAs include the chemoenzymatic method, chemical method, and ribozyme method (Figure 5). In the chemoenzymatic method, the first step is the chemical acylation of hybrid dinucleotide 5′-phospho-2′-deoxyribocytidylylriboadenosine (pdCpA) using an activated amino acid donor with an N-protected group. In the following step, the acylated dinucleotide is enzymatically ligated via T4 ligase to truncated tRNA lacking 3′-CA dinucleotide (Hecht et al., 1978). Many functional groups can be ligated to tRNA with this approach, but it is cumbersome due to the laborious chemical process involved in the preparation of acylated nucleotides.

In the chemical method, the side chains of the amino acids are chemically modified after tRNAs were charged with canonical amino acids by the AARS. One can consider this method similar to post-translational modification of proteins; instead, here it is the charged tRNA that is modified by a chemical reaction. Diverse functional groups such as N-methyl amino acid (Merryman and Green, 2004), glycosyl amino acid (Fahmi et al., 2007), and fluorescently-labeled amino acids (Iijima and Hohsaka, 2009) were generated with this approach. However, similar to the chemoenzymatic method, this method is laborious, technically demanding, and suffers from poor efficiency due to cyclic-tRNA by-product which inhibits protein synthesis, and also the short lifetime of aminoacylated tRNA (Yamanaka et al., 2004).

Another approach for incorporating NC-AA that avoids the use of AARS altogether is performed by ribozymes called flexizymes pioneered by Prof. Hiroaki Suga. A flexizyme is a small artificial ribozyme (44–46 nucleotides) capable of generating NC-AA tRNAs (Murakami et al., 2006). Flexizyme is derived from acyl-transferase ribozyme through directed evolution and sequence optimization. Flexizyme specifically recognizes amino acids with an activated carboxyl group and charge the 3′-CCA end of tRNA irrespective of tRNA body and anticodon sequence (Xiao et al., 2008). Flexizyme classes were expanded lately such that they can accept amino acids with different active groups. The complete set of flexizymes includes dinitro flexizyme (dFx) recognizing amino acid with activated dinitrobenzyl ester, enhanced flexizyme (eFx) recognizing amino acid with chlorobenzyl ester, and amino flexizyme (aFx) recognizing amino acid with the amino derivatized benzyl thioester group (Morimoto et al., 2011). Flexizymes can accept almost all amino acids as acyl-donor substrates and expand the diversity of amino acids that can be incorporated. For instance, flexizyme-mediated incorporation of NC-AA with D-α-amino acids (Katoh, Tajima, and Suga, 2017), β-amino acids (Katoh and Suga, 2018), γ-amino acids (Ohshiro et al., 2011), N-alkyl-L- α-amino acids (Kawakami, Ishizawa, and Murakami, 2013), benzoic acids (Kawakami et al., 2016), and exotic peptides (Goto and Suga, 2009) to highlight a few. Aminoacylation is performed by incubating the desired tRNA and amino acid together to yield charged tRNA (Goto, Katoh, and Suga, 2011). The only limitation that exists from the amino acid is the ability to activate the carboxyl group with a certain group and the chemical stability of amino acids during aminoacylation. This technique has opened up the possibility to charge any tRNA with nearly any amino acid and thereby reassigning any codon with NC-AAs. The *in vitro* translation system based on flexizyme and translation apparatus is called the flexible *in vitro* translation system (FIT) and has been used for genetic code reprogramming (Goto, Katoh, and Suga, 2011).

## 5 Challenges and Future Directions

Protein synthesis is a highly complex process with many players involved that ensure fidelity. The process of translation has evolved over billions of years to attain the current state of efficiency and quality control. Attempts to modify, alter, or improve the efficiency of translation to suit a novel application require efforts at multiple levels *in vivo* to reach the desired function. CFPS systems eliminate some of the stringency associated with *in vivo* translation and simplified engineering of the protein synthesis process to some extent. For genetic code expansion, CFPS has opened new avenues for incorporating a diverse range of NC-AAs and helped achieve proteins with novel functional groups. The development of an orthogonal translation system requires a coordinated effect at every level of protein expression. Apart from tRNAs and AARSs engineering, engineering efforts on other translational elements such as elongation factors (Fan, Ip, and Söll, 2016; Gan et al., 2017), initiation factors (Goto et al., 2008; Goto and Suga, 2009), ribosomes (Semrad and Green, 2002; Jewett et al., 2013; Li et al., 2017a), and termination factors (Hong et al., 2015; Korkmaz and Sanyal, 2017) helped in the development of a better translational system. One key challenge lies in bringing together these individually engineered components. CFPS research will continue to increase our understanding and the capability to engineer the improved translational system. Failures will reveal the gaps in our understanding and guide our scientific research. Despite ongoing effects, many areas require advancements. For instance, the efficiency of NC-AA is low due to the non-compatibility of structurally diverse NC-AA with ribosomes and elongation factors (Fujino et al., 2013). This requires better engineered translation factors to successfully incorporate NC-AA with high efficiency. The advent of high-throughput methods and computational techniques like MAGE and PACE quickened the pace of library generation and screening, leading to faster protein engineering. GCE has increased amino acid repertoires with diverse functional and structural groups and has resulted in the creation of novel proteins that could not be synthesized before. The use of such novel proteins could be in creating better drugs, and functional biomaterials. Improvements and better characterization of translation elements, such as AARS and tRNA, using the latest techniques will help to standardize translation elements and aid in creating a predictable biological system. From a synthetic biology perspective, engineering life from scratch remains a grand challenge in the field. Toward that goal, different aspects of living systems have been reconstituted *in vitro* including ATP synthesis (Berhanu, Ueda, and Kuruma, 2019), DNA replication (Kurihara et al., 2011), PURE protein component self-regeneration (Lavickova, Laohakunakorn, and Maerkl, 2020), and ribosomal components (Jewett et al., 2013). With respect to the PURE system, the efficiency is improved by addition of external components into the system such as EF-P, EF4, and ArfA (Li et al., 2017b). Cell-free transcription and translation systems have enormous potential to overcome the limits of cell-based protein synthesis and could become the next generation platform for protein engineering that can go well beyond the scope of what could be accomplished in a cellular environment.
